# Collectanea Medica: Consisting of Anecdotes, Facts, Extracts, Illustrations, &c.

**Published:** 1821-07

**Authors:** 


### COLLECTANEA MEDICA:

CONSISTING OF

### ANECDOTES, FACTS, EXTRACTS, ILLUSTRATIONS, &c.

*Relating to the History or the Art of Medicine, and the Auxiliary Sciences.*

Floriferis ut apes in saltibus omnia libant,

Omnia nos itidem depascimur aurea dicta.

---

*Extracts of the Report from the Select Committee of the House of Commons on the Doctrine of the Contagion of the Plague, in* 1819.

*Jovis*, 13° *die Maij*, 1819.

Sir John Jackson, Baronet, in the Chair.

James Frank, M.D. called in; and Examined.

HAVE you been in countries where the plague existed?—Yes, I have.

What is your opinion of its contagious effects?—That it is highly contagious.

How do you form that opinion?—I was upon the expedition with Sir Ralph Abercrombie, in the year 1800, and had the first establishment of the plague-hospital at Aboukir. The army landed in the month of March, and was perfectly free from the disease till about the middle of May; when a person from the commissariat depot at Aboukir was reported to be ill, and it was said that he had the plague: he was removed from thence to the hospital, which might be the distance of about a mile, and confined in a tent by himself. He had been ill about four-and-twenty hours before at the depot, and it was supposed that he was intoxicated, that his disease arose from excess: he died, I think, on the second or third day. Two more persons, from the same depôt, in a day or two afterwards, were reported to be sick in the same manner, and they were sent to the same place. It was doubtful whether it was the disease; and then it became a question how it had got to the depôt. There were two reports: one, that it was imported by a Greek boat from Cyprus, with Cyprus wine; and the other, that it was brought from Rhamaneh by the Arabs, where it was known the plague had been raging. It was afterwards said that a man had died on-board this Greek boat. The impression upon my mind is, that it was brought by this Greek boat: the disease had been at Cyprus and upon the coast of Syria; and Brigadier-General Koëhler, and the detachment of artillery under his command, had fallen victims to the disease, and died at Jaffa three months previous. I have stated that I had the superintendance of the plague-hospital at Aboukir, and consequently the arrangement for the accommodation of the plague-patients, though not the immediate charge of the sick; for it had spread from the attendants who had caught the disease from those persons above mentioned, who had died, to the sick of the hospital. My whole arrangements were made, upon the principle of its being contagious, to prevent its spreading to the army, which was in the lines before Alexandria, at the distance of ten or twelve miles from the depôt. The hospital at that time consisted of the wounded men after the actions of the 8th, the 13th, and the 21st of March. There were very few sick, who were placed in some rude huts which the French had built; for there was no house whatever at Aboukir, the village having been destroyed; and among those sick the plague-patients were sent, it having appeared that the disease had been communicated to them by the attendants upon the first plague-patients; and, as it was impossible to say who had got the plague or who had not, I recommended that the whole should be guarded by sentinels, and that the whole should be thrown into quarantine: this was effected. The disease increased in the huts at Aboukir; and the medical officers, and the servants attending upon those patients, were almost all seized with the disease, and several of them died; three or four hospital-mates died. From these facts I should state, that it is my opinion that it is a contagious disease; for, had it not been so, there would have been an equal number of patients, or probably more, among the wounded than there were in the huts; the wounded men, who were in tents and in temporary buildings, being separated from the huts at least half a mile, or probably nearer a mile. These precautions having been taken, of throwing the whole of the plague-patients, as soon as circumstances would admit, under quarantine, I think, kept the army upon the lines before Alexandria very free from the disease; for I believe there were very few, if any, cases of plague sent from thence to Aboukir.

Had any of the army at Aboukir the plague?—The plague was almost entirely confined to Aboukir: there could have been but very few, if any, of the army before Alexandria who had the plague during the whole of the time that the plague-hospital was at Aboukir.

Have you any idea of the number of military that took the plague? —I have no return, my papers were all lost; but I should think that return might be got from the army medical board: I should think about three or four hundred; they were chiefly from the sick in the huts, chiefly among the patients and servants. I do not recollect any cases that were sent from the lines; they were chiefly confined to the peninsula of Aboukir, and principally among the huts.

How long did the hospital remain at Aboukir?—I think it was broken up in July.

Were there any plague-cases in the hospital at that time?—I believe there were two or three; they were very trifling cases; I think they were sent to Rosetta. I was at Rosetta at the time it was broken up.

Were there a great many deaths?—I do not know what was the proportion of deaths; I should think about one in four, but I do not speak from certain knowledge: that is the impression upon my mind.

Did you happen to hear of any cases of plague having occurred in the army that advanced to Cairo?—There were plague-hospitals established at Rosetta; and I imagine those cases must have been sent from the army which advanced to Cairo, or from the troops which remained at Rosetta.

Have you any knowledge of the disease originating from any local circumstances in Egypt?—No, I have not.

Is it not considered to be endemic in that country?—It is epidemic and endemic, both; that is to say, there are particular places where it rages more especially. From what I understand, it does not break out every year, or every two years, or every three years.

What do you suppose to be the cause of the plague?—I do not know the cause; but the plague itself appears to me to be a specific contagion, that is brought into action by some particular constitution of air.

Do you suppose that its revival annually is an originating, or that it depends upon former circumstances?—That is very difficult to say. Probably it is conjecture that it depends upon a certain constitution of air to bring it into action; but it is very sure that there is a certain range of temperature upon the thermometer that is necessary for its appearance. The great cold of Constantinople puts an end to it, and the great heat of Cairo puts an end to it.

That goes to the termination of it?—Yes; but the same applies to the beggining of it; for it never begins in great cold nor great heat.

Does it begin as a new revival, a new origin, or is a seed or virus latent brought into action by circumstances of air or other circumstances. Do you suppose the seeds of it remain perpetually?—Yes; and that it is brought into action by a particular constitution of air.

How do you account for the expurgators of goods in our quarantine establishments not having had the plague for so long a period?—It is a certain fact that the plague is destroyed by ventilation; it is also certain that it exists only in a particular constitution of air. I can very readily imagine that the constitution of air required is not present, though the seeds of the disease may be present, and that it is not called into action. Whether the constitution of the air renders the body susceptible of taking the disease or not, is a very difficult question, or one impossible, perhaps, to answer.

Would you consider that its not having been called into action by any thing whatever, at our quarantine establishments, for upwards of a hundred years, there is ground to conclude that the quarantine establishments are useless?—No.

Do you consider that it is equally communicable from goods as from persons?—Equally.

As there have been no persons arriving at the quarantine establishments having the plague, is it not fair, from that circumstance, to conclude that the infection has not arrived in goods?—I stated that I thought a particular constitution of the air was absolutely necessary: what that may be, I know not; but I perceive that the disease rages under a certain temperature, or in a particular state of atmosphere. I conceive that that is not present. I suppose that if the goods were infected when they were put on-board at Smyrna, or any other place, they would arrive in the same state here; because I do not see how those goods can be ventilated whilst they are in bales, nor see how they can be exposed to air upon the passage.

If a person infected with the plague on-board any vessel in the Mediterranean, was coming to Great Britain, should you suppose that he would either die or become cured, for want of that proper and appropriate state of the air?—He would die or be cured upon the passage, if the voyage was very long: if he lived, he would probably arrive without the seeds of the disease about him.

From no person having arrived infected with the plague for so many years, do you not consider that it is almost impossible for a person to reach our quarantine establishments with the plague present in him at the moment of his arrival?—I think the length of the voyage, as to persons, would sufficiently purify them.

And cure them?—Yes. If the disease manifested itself, it would go through its course, and the person would recover or die; but, if it did not show itself, I do not believe the disease would remain latent so long.

Perhaps you consider that it would not be possible to introduce a person from the Mediterranean infected with the plague, into our quarantine establishments, in consequence of the length of voyage?—After a long voyage, I should certainly say not. I only speak with respect to the persons, and to their clothes which have been exposed to the air during the voyage.

Your opinion of such security is confined entirely to the persons and their clothes exposed to the air, but has no reference to the circumstance of their touching any goods on-board that may have the infection?—Certainly.

Is it your opinion that a cargo of goods might arrive from the Levant in this country, and bulk not being broken, should be again carried back to the Levant, and then, on returning to that state of atmosphere which calls into action that disease, might affect the inhabitants of that country, though it would not have infected the inhabitants of this country if the bales had been opened?—That is a very difficult question to answer; because, though the state of the atmosphere in the Levant might be favourable to bringing into action the contagion, yet it might not be so here: and that that state of the atmosphere is necessary, is very obvious, because it is only at certain times that the disease shows itself in those countries, at Smyrna, at Aleppo, at Grand Cairo, and at other places.

Would the conveying infected goods into a climate less favourable to the spreading of the disease, not destroy the infection so as to render them incapable of conveying the infection, even under circumstances the most favourable for the spreading of the disease; for instance, in the Levant?—I think it probable that infected goods will retain the infection, unless exposed to the air; and therefore, whenever they are exposed to the air, the persons handling them are liable to infection, if certain circumstances are favourable to bringing out the contagion.

Do you believe that the plague ever did exist in this climate?—Yes.

The true Levant plague?—The true Levant plague. I believe it did in the year 1665.

Is it your opinion that there must have existed some peculiarity in the state of the atmosphere at that period, which was favourable to the spreading of the contagion?—I think there must have been some concurring causes which brought it into activity: the atmosphere alone can never produce the plague. The plague, I have stated before, is a specific contagion, producing a specific effect upon the body.

Although no instance has occurred in this country of the plague since the year 1665, is it safe to conclude that circumstances might not arise which might be conducive to the spreading of that infection?—I think that circumstances might arise that would be conducive to the spreading of the infection.

Is your opinion that the quarantine is necessary, founded on the chance of such a recurrence, or on any other cause?—Certainly founded on the chance of such a recurrence.

Do you consider the plague of 1665 to have been imported into England?—The impression on my mind is, that it was imported.

Do you consider the cause of the plague not having appeared in our quarantines, to have probably occurred from the infection not having arrived, or to the attention paid to ventilating ships arriving from the Mediterranean?—Very probably to the excellent mode of ventilation.

And not so much the annihilation of the infection, from the length of the voyage?—No, certainly not.

Have you not often heard of cases of persons who have been in close contact with plague-patients, never having taken the plague?—Yes, I have: I myself am one; I have frequently touched plague-patients, and never had the disease.

Then it is not so active a contagion as small-pox?—The contagion of the small-pox, when it produces the confluent disease, is often as fatal as that of the plague: it exists in a state of activity in tropical climates, which I believe the plague never does. The plague varies at different times in Egypt: I speak of the plague which I saw in the country in which I was. When it first breaks out, which is about the month of January or February, the cases are few, but they are more violent in their symptoms: as the season goes on, the disease becomes milder, but more frequent, in consequence of a greater number of persons being exposed to the infection; it then declines, and ceases altogether at the summer solstice.

Does it cease about the 24th of June invariably?—At Cairo; but it is sporadic upon the coast in July, after the summer solstice.

Do you consider it more contagious when it is the most violent?—Yes, I do; certainly.

Have you known any instances of medical men inoculating themselves for the plague?—Yes, I have; Dr. White.

Did he die of the complaint?—I believe he died of the complaint, but that is uncertain: he never would suffer himself to be examined whilst he had the disease. It was known he had inoculated himself with matter taken from a pestilential bubo; that he had a shivering-fit, and was frightened; that he got up at night and washed his wrist with some volatile alkali, with the hopes of preventing absorption: but he died in the course of three or four days afterwards.

Whether of the plague or not, you are not certain?—I do not know whether he had buboes or not: I should say, but it is merely from hearsay, that he did die of the plague. He never would suffer himself to be examined by the medical men while he had the disease.

Was his body examined after his death?—I believe not.

Were you acquainted with Dr. White?—Yes.

Have you heard him give his opinion respecting the plague?—Yes, I have.

What was that opinion?—That it was not contagious.

